# Supplementary material for: Content-rich biological network constructed by mining PubMed abstracts
Source: BMC Bioinformatics. 2004 Oct 8;5:147. doi: 10.1186/1471-2105-5-147 (PMC528731; doi:10.1186/1471-2105-5-147)
Supplement: Additional File 2 — The original results of the above study (non-essential files are deleted to keep the file size under the limit set by BMC bioinformatics). [file 1471-2105-5-147-S2.bz2 › chilibotAdditionalFile2/dip20/583ID9533885E10951/html/NUTF2_RAN.html]

 


 **NUTF2** and **RAN** 
  
Found 47 abstracts in PubMed, retrieved 20.  
 

 What does Google say? 
 PDF only 
| .edu only 

---

**Interactive relationship** (e.g. stimulation, inhibition, etc)

**Neutral relationship**- While transport factors such as  **Ran**  and NTF2  [ **NUTF2** ]  show only transient interaction with the pores, a number of soluble transport receptors, including importin beta, show a tight association with the purified pores.  Ref: 11208084 Traffic, 2000
- The interaction between  **Ran**  and NTF2  [ **NUTF2** ]  is required for cell cycle progression.  Ref: 10930458 Mol Biol Cell, 2000
- This provides the first evidence that NTF2  [ **NUTF2** ]  regulates the distribution of  **Ran**  in vivo.  Ref: 10679025 Mol Biol Cell, 2000
- These findings demonstrate that NTF2  [ **NUTF2** ]  is required to transport  **Ran**  into the nucleus in vivo.  Ref: 10930458 Mol Biol Cell, 2000
- A temperature sensitive ts mutant of Saccharomyces cerevisiae NTF2  [ **NUTF2** ]  that does not bind to  **Ran**  is unable to import  **Ran**  into the nucleus at the nonpermissive temperature.  Ref: 10930458 Mol Biol Cell, 2000
- The interaction between NTF2  [ **NUTF2** ]  and  **Ran**  is essential for the import of  **Ran**  into the nucleus.  Ref: 11489893 J Biol Chem, 2001
- Here we show that cytoplasmic injection of anti NTF2  [ **NUTF2** ]  mAbs resulted in a dramatic relocalization of  **Ran**  to the cytoplasm.  Ref: 10679025 Mol Biol Cell, 2000
- We show that nuclear transport factor 2  [ **NUTF2** ]  NTF2  [ **NUTF2** ]  is required to concentrate  **Ran**  in the nucleus in the budding yeast, Saccharomyces cerevisiae.  Ref: 10889207 J Biol Chem, 2000
- NTF2  [ **NUTF2** ]  mediated import of  **Ran**  appears to be the bottleneck for maximal capacity of  **Ran**  driven nuclear transport.  Ref: 12606574 EMBO J, 2003
- Evidence that NTF2  [ **NUTF2** ]  directly mediates  **Ran**  import or that NTF2  [ **NUTF2** ]  is required to maintain the nuclear concentration of  **Ran**  in living cells has not been obtained.  Ref: 10679025 Mol Biol Cell, 2000
- Taken together, our data show that NTF2  [ **NUTF2** ]  is an essential regulator of the  **Ran**  distribution in living cells.  Ref: 10679025 Mol Biol Cell, 2000
- NTF2  [ **NUTF2** ]  has been shown to bind nuclear pore complex proteins and the GDP form of  **Ran**  in vitro.  Ref: 10679025 Mol Biol Cell, 2000
- Proteins that regulate the GTPase cycle and subcellular distribution of  **Ran**  include the cytoplasmic GTPase activating protein RanGAP and its co factors RanBP1, RanBP2, the nuclear guanine nucleotide exchange factor RanGEF, and the  **Ran**  import receptor NTF2  [ **NUTF2** ] .  Ref: 10811801 J Biol Chem, 2000
- Engineered mutants at the interaction interface reduce the binding of NTF2  [ **NUTF2** ]  to nuclear pores and cause reduced growth rates and  **Ran**  mislocalization when substituted for the wild type protein in yeast.  Ref: 12065398 EMBO J, 2002
- Recently, it has been reported that NTF2  [ **NUTF2** ]  can stimulate the accumulation of  **Ran**  in digitonin permeabilized cells.  Ref: 10679025 Mol Biol Cell, 2000
- Monoclonal antibodies to NTF2  [ **NUTF2** ]  inhibit nuclear protein import by preventing nuclear translocation of the GTPase  **Ran** .  Ref: 10679025 Mol Biol Cell, 2000
- Moreover, anti NTF2  [ **NUTF2** ]  mAbs inhibited nuclear import of both  **Ran**  and NLS containing protein in vitro, suggesting that NTF2  [ **NUTF2** ]  stimulates NLS dependent protein import by driving the nuclear accumulation of  **Ran** .  Ref: 10679025 Mol Biol Cell, 2000

**Non-interactive relationship** (e.g. studied together, co-existance, homology, etc.)

- The mechanism of  **ran**  import into the nucleus by nuclear transport factor 2  [ **NUTF2** ] .  Ref: 10889207 J Biol Chem, 2000
- Our data distinguish between two possible mechanisms for  **Ran**  import by NTF2  [ **NUTF2** ]  and demonstrate that  **Ran**  import is independent from importin beta mediated protein import.  Ref: 10889207 J Biol Chem, 2000
- Here, we examine the in vivo role of NTF2  [ **NUTF2** ]  in  **Ran**  import and the effect that disruption of  **Ran**  imported into the nucleus has on the cell cycle.  Ref: 10930458 Mol Biol Cell, 2000
- The TAP p15 heterodimer is structurally similar to the homodimeric transport factor NTF2  [ **NUTF2** ] , but unlike NTF2  [ **NUTF2** ] , it is incompatible with either homodimerization or  **Ran**  binding.  Ref: 11583626 Mol Cell, 2001
- In contrast, a significant reduction in affinity of the RanGDP binding protein NTF2  [ **NUTF2** ]  for  **Ran**  CoreGDP is observed, indicating that the switch regions have changed conformation in the  **Ran**  Core mutant.  Ref: 12051861 J Mol Biol, 2002
- Consistent with a critical role for NTF2  [ **NUTF2** ]  in establishing and maintaining the  **Ran**  gradient, we show that NTF2  [ **NUTF2** ]  is required for early embryogenesis in Caenorhabditis elegans.  Ref: 10889207 J Biol Chem, 2000
- Four mutant complementation groups were characterized that correspond to respective mutations in genes encoding  **Ran**  gsp1, and essential  **Ran**  regulatory factors  **Ran**  GTPase  [ **RAN** ]  activating protein rna1,  **Ran**  guanine nucleotide exchange factor prp20, and the RanGDP import factor ntf2  [ **NUTF2** ] .  Ref: 12654904 J Cell Biol, 2003
- To analyze the mechanism of  **Ran**  import into the nucleus by NTF2  [ **NUTF2** ] , we use mutants in a variety of nuclear transport factors along with biochemical analyses of NTF2  [ **NUTF2** ]  complexes.  Ref: 10889207 J Biol Chem, 2000
- Surprisingly, we found that neither Xenopus ovarian cytosol nor a mixture of recombinant import factors karyopherin alpha2, karyopherin beta1,  **Ran** , and p10 NTF2  [ **NUTF2** ]  were able to support the import of pA RCC1 into the nuclei of digitonin permeabilized cells.  Ref: 10744690 J Biol Chem, 2000
- 4 Importin beta1,  **Ran** , and p10 NTF2  [ **NUTF2** ]  are sufficient to mediate import of activated Smad3.  Ref: 11294908 Mol Biol Cell, 2001
- NTF2  [ **NUTF2** ]  mediated  **Ran**  nuclear import is required for NLS dependent protein import.  Ref: 10679025 Mol Biol Cell, 2000
- We find that  **Ran**  remains concentrated in the nucleus when importin mediated protein import is disrupted and demonstrate that NTF2  [ **NUTF2** ]  does not form a stable complex with the transport receptor, importin beta.  Ref: 10889207 J Biol Chem, 2000
